# Coronary Calcified Nodules: From Pathological Definitions to Intravascular Imaging- and Morphology-Guided PCI

**DOI:** 10.3390/ijms27156999

**Published:** 2026-08-04

**Authors:** Mateusz Lucki, Sylwia Iwańczyk, Ewa Lucka, Marek Grygier, Przemysław Mitkowski, Maciej Lesiak

**Affiliations:** 1 Department and Clinic of Cardiology, University of Medical Sciences, 60-545 Poznań, Poland; mateusz.lucki@ump.edu.pl (M.L.); siwanczyk@ump.edu.pl (S.I.); marek.grygier@ump.edu.pl (M.G.); przemyslaw.mitkowski@ump.edu.pl (P.M.); maciej.lesiak@ump.edu.pl (M.L.); 2 Clinical Rehabilitation Laboratory, Department of Rehabilitation and Physiotherapy, University of Medical Sciences, 60-545 Poznań, Poland

**Keywords:** calcified nodules, coronary artery calcification, coronary artery disease, intravascular imaging, intravascular ultrasound, nodular calcium, optical coherence tomography, percutaneous coronary intervention

## Abstract

Coronary artery calcification (CAC) is a hallmark of advanced atherosclerosis and a major determinant of procedural complexity during percutaneous coronary intervention (PCI). Once considered a passive consequence of vascular degeneration, CAC is now recognized as an active, highly regulated process driven by inflammation, oxidative stress, extracellular vesicle release, osteogenic differentiation of vascular smooth muscle cells, and biomechanical remodeling. These mechanisms generate a spectrum of calcific phenotypes, ranging from microcalcifications and sheet calcium to nodular calcium and calcified nodules. Calcified nodules represent an advanced fibrocalcific plaque phenotype characterized by fractured calcific plates, luminal calcium protrusion, surface disruption, and variable thrombus formation. They can be characterized using intravascular ultrasound (IVUS), optical coherence tomography (OCT), and hybrid near-infrared spectroscopy–IVUS imaging, and are associated with coronary thrombosis, stent underexpansion, restenosis, target lesion failure, and the need for advanced calcium-modification strategies. A structured literature search of PubMed/MEDLINE, Web of Science Core Collection, and Scopus identified 83 publications published between 2020 and 2026 for inclusion in the narrative synthesis. This narrative review summarizes the biological and biomechaniclam mechanisms of coronary calcification and calcified nodule formation, compares multimodality intravascular imaging criteria, and discusses contemporary imaging-guided PCI strategies, including balloon-based modification, rotational and orbital atherectomy, excimer laser coronary atherectomy, intravascular lithotripsy, and hybrid approaches. By integrating pathobiology, intravascular imaging criteria, and lesion-specific PCI strategies, this review provides a clinically oriented framework for the assessment and management of calcified nodules. Future directions include standardized imaging definitions, prospectively validated morphology-guided treatment algorithms, and computational and artificial intelligence-assisted plaque characterization.

## 1. Introduction

Coronary artery calcification (CAC) is a hallmark of advanced atherosclerotic disease and an increasingly common finding in contemporary interventional cardiology practice [1,2]. As the number of complex coronary interventions continues to increase, heavily calcified lesions remain among the most challenging anatomical subsets encountered during percutaneous coronary intervention (PCI) [3]. Their prevalence continues to rise owing to population aging and the growing burden of diabetes mellitus, chronic kidney disease, and metabolic disorders [4,5]. Consequently, coronary calcification has become a major determinant of procedural complexity, contributing to difficulties in lesion crossing, device delivery, stent expansion, and long-term clinical outcomes [6,7].

Traditionally, vascular calcification was regarded as a passive consequence of aging and plaque degeneration. However, accumulating evidence indicates that coronary calcification is a highly regulated biological process involving chronic inflammation, endothelial dysfunction, oxidative stress, extracellular vesicle release, and osteogenic differentiation of vascular smooth muscle cells [8,9,10]. These mechanisms share important similarities with physiological bone formation and promote progressive calcium deposition within the arterial wall. Accordingly, coronary calcification is now recognized not only as a marker of advanced atherosclerosis but also as an active contributor to plaque progression, vascular remodeling, and adverse cardiovascular outcomes [11,12].

Importantly, coronary calcification is a heterogeneous process. Histopathological and intracoronary imaging studies have identified several distinct calcific phenotypes, including microcalcifications, spotty calcification, sheet calcium, nodular calcium, and calcified nodules [13]. These phenotypes differ substantially in their biological behavior, biomechanical properties, and clinical implications [14]. While extensive sheet calcium has generally been associated with plaque stabilization, smaller and structurally heterogeneous calcific deposits may promote plaque vulnerability, alter biomechanical stress distribution, and contribute to adverse cardiovascular events [15,16].

Among these phenotypes, calcified nodules have emerged as a particularly intriguing and clinically relevant manifestation of advanced coronary artery disease. First described by Virmani and colleagues, calcified nodules are characterized by fragmented calcific plates and protruding nodular calcium extending into the vessel lumen, frequently accompanied by endothelial disruption and thrombus formation [17]. Although they account for a smaller proportion of acute coronary syndromes than plaque rupture or plaque erosion, increasing use of high-resolution intracoronary imaging has demonstrated that these lesions are more prevalent than previously appreciated, particularly among elderly patients and those with severe coronary calcification, diabetes mellitus, or chronic kidney disease [18,19,20]. Growing evidence suggests that calcified nodules represent not merely a pathological curiosity but a distinct lesion phenotype associated with unique biological, diagnostic, prognostic, and procedural challenges.

Beyond their role in coronary thrombosis and acute coronary syndromes, calcified nodules have attracted considerable attention because of their association with adverse PCI outcomes. Previous studies have demonstrated that nodular calcium and calcified nodules are linked to stent underexpansion, incomplete lesion preparation, restenosis, stent thrombosis, and target lesion failure [21,22]. Consequently, accurate identification and characterization of these lesions have become increasingly important for procedural planning and optimization.

The emergence of contemporary intravascular imaging has fundamentally transformed the assessment of coronary calcification. While conventional angiography frequently underestimates calcium burden and provides limited information regarding plaque morphology, intravascular ultrasound (IVUS), optical coherence tomography (OCT), and near-infrared spectroscopy (NIRS) enable comprehensive characterization of calcium distribution, arc, thickness, plaque composition, and lesion vulnerability [23,24,25]. These modalities provide complementary rather than competing information, facilitating a more comprehensive assessment of lesion morphology and procedural risk. As a result, intravascular imaging has become central to imaging-guided PCI and plays a critical role in selecting appropriate calcium-modification strategies, including rotational atherectomy (RA), orbital atherectomy (OA), excimer laser coronary atherectomy (ELCA), and intravascular lithotripsy (IVL) [26,27].

Despite substantial advances in the understanding of coronary calcification, important uncertainties remain regarding the relationship between the biological evolution of fibrocalcific plaques, the pathological definition of calcified nodules, their appearance on contemporary intravascular imaging, and the selection of lesion-specific treatment strategies. These uncertainties are clinically relevant because the terms nodular calcium, non-eruptive calcified nodule, and eruptive calcified nodule are not consistently defined or applied across pathological, OCT, and IVUS studies, thereby complicating comparisons among investigations and the translation of imaging findings into procedural decision-making [3,18,22].

Several recent reviews have addressed coronary calcification from complementary perspectives. Broad state-of-the-art reviews have summarized invasive and non-invasive imaging techniques, lesion-preparation technologies, and treatment algorithms for calcified coronary stenosis [28,29], whereas calcified nodule-focused reviews have primarily examined their prevalence, eruptive and non-eruptive subtypes, prognostic significance, and therapeutic challenges [30,31]. A recent systematic review has additionally evaluated the prevalence and clinical associations of calcified nodules and their impact on procedural and long-term outcomes following PCI [32]. However, these publications have generally focused either on the overall management of calcified coronary lesions, on the clinical outcomes of calcified nodules, or on selected aspects of their imaging and treatment.

The intended added value of the present review is a structured, lesion-specific synthesis that places the pathology–imaging discrepancy at the centre of the discussion and links the biological continuum of coronary calcification with multimodality imaging criteria and morphology-guided PCI strategies. Specifically, we trace the biological and biomechanical transition from sheet calcium to nodular calcium and calcified nodules; explicitly examine the discrepancy between pathological definitions and imaging-based classifications; compare the complementary diagnostic contributions and limitations of OCT, IVUS, and NIRS; and translate lesion morphology into practical calcium-modification strategies Particular emphasis is placed on the distinction between smooth nodular calcium and irregular or eruptive calcified nodules, the presence of surface disruption or thrombus, calcium depth and circumferential distribution, and the implications of these features for balloon-based modification, RA, OA, ELCA, IVL, and hybrid approaches. Thus, rather than providing another general overview of coronary calcification, this review offers a lesion-specific framework linking biological evolution and diagnostic classification with morphology-guided PCI.

Accordingly, this narrative review first examines the biological and biomechanical mechanisms of coronary calcification and calcified nodule formation. It then addresses the pathological and clinical significance of calcified nodules, compares the complementary roles of IVUS, OCT, and NIRS, and translates lesion morphology into contemporary imaging-guided PCI strategies.

## 2. Review Methodology

This narrative review was prepared in accordance with the SANRA (Scale for the Assessment of Narrative Review Articles) recommendations [33]. Its objective was to synthesize contemporary evidence on the biological and biomechanical mechanisms of coronary calcification, the pathobiology and clinical significance of calcified nodules, their characterization using intravascular imaging, and contemporary morphology-guided percutaneous coronary intervention (PCI) strategies.

A comprehensive literature search was conducted in PubMed/MEDLINE, Web of Science Core Collection, and Scopus, covering peer-reviewed publications published in English between 1 January 2020 and 29 July 2026. Reference lists of relevant original studies, systematic reviews, meta-analyses, expert consensus documents, and clinical practice statements were also manually screened to identify additional eligible publications.

The complete PubMed/MEDLINE search syntax was as follows: ((“coronary artery calcification”[Title/Abstract] OR “coronary calcification”[Title/Abstract] OR “calcified coronary lesion*”[Title/Abstract] OR “calcified nodule”[Title/Abstract] OR “calcified nodules”[Title/Abstract] OR “nodular calcium”[Title/Abstract] OR “fibrocalcific plaque”[Title/Abstract]) AND (mechanism*[Title/Abstract] OR pathobiolog*[Title/Abstract] OR histopatholog*[Title/Abstract] OR biomechanic*[Title/Abstract] OR “intravascular imaging”[Title/Abstract] OR “intracoronary imaging”[Title/Abstract] OR “intravascular ultrasound”[Title/Abstract] OR IVUS[Title/Abstract] OR “optical coherence tomography”[Title/Abstract] OR OCT[Title/Abstract] OR “near-infrared spectroscopy”[Title/Abstract] OR NIRS[Title/Abstract] OR “percutaneous coronary intervention”[Title/Abstract] OR PCI[Title/Abstract] OR “calcium modification”[Title/Abstract] OR “rotational atherectomy”[Title/Abstract] OR “orbital atherectomy”[Title/Abstract] OR “excimer laser coronary atherectomy”[Title/Abstract] OR ELCA[Title/Abstract] OR “intravascular lithotripsy”[Title/Abstract] OR IVL[Title/Abstract] OR “scoring balloon”[Title/Abstract] OR “cutting balloon”[Title/Abstract])) AND (“2020/01/01”[Date-Publication]: “2026/07/29”[Date-Publication]) AND English[Language].

The Web of Science Core Collection search syntax was:TS=((“coronary artery calcification” OR “coronary calcification” OR “calcified coronary lesion*” OR “calcified nodule*” OR “nodular calcium” OR “fibrocalcific plaque*”) AND (mechanism* OR pathobiolog* OR histopatholog* OR biomechanic* OR “intravascular imaging” OR “intracoronary imaging” OR “intravascular ultrasound” OR IVUS OR “optical coherence tomography” OR OCT OR “near-infrared spectroscopy” OR NIRS OR “percutaneous coronary intervention” OR PCI OR “calcium modification” OR “rotational atherectomy” OR “orbital atherectomy” OR “excimer laser coronary atherectomy” OR ELCA OR “intravascular lithotripsy” OR IVL OR “scoring balloon*” OR “cutting balloon*”)) AND PY=(2020–2026) AND LA=(English).

The Scopus search syntax was: TITLE-ABS-KEY((“coronary artery calcification” OR “coronary calcification” OR “calcified coronary lesion*” OR “calcified nodule*” OR “nodular calcium” OR “fibrocalcific plaque*”) AND (mechanism* OR pathobiolog* OR histopatholog* OR biomechanic* OR “intravascular imaging” OR “intracoronary imaging” OR “intravascular ultrasound” OR IVUS OR “optical coherence tomography” OR OCT OR “near-infrared spectroscopy” OR NIRS OR “percutaneous coronary intervention” OR PCI OR “calcium modification” OR “rotational atherectomy” OR “orbital atherectomy” OR “excimer laser coronary atherectomy” OR ELCA OR “intravascular lithotripsy” OR IVL OR “scoring balloon*” OR “cutting balloon*”)) AND PUBYEAR > 2019 AND PUBYEAR < 2027 AND (LIMIT-TO(LANGUAGE, “English”)).

Eligible publications included randomized clinical trials, prospective and retrospective clinical studies, intracoronary imaging studies, registries, histopathological and translational investigations, systematic reviews, meta-analyses, expert consensus documents, and clinical practice statements. Studies were required to address at least one of the following domains: mechanisms of coronary calcification; morphology and clinical significance of calcified nodules; assessment using IVUS, OCT, NIRS, or hybrid imaging; or imaging-guided PCI and calcium-modification strategies.

Case reports, small anecdotal case series, animal or in vitro studies without direct translational relevance, conference abstracts, editorials, letters without substantive original data, non-peer-reviewed publications, non-English-language articles, and studies not directly related to the scope of the review were excluded. Overlapping or superseded reports were excluded when a more complete or updated publication from the same study was available.

**Figure 1:**
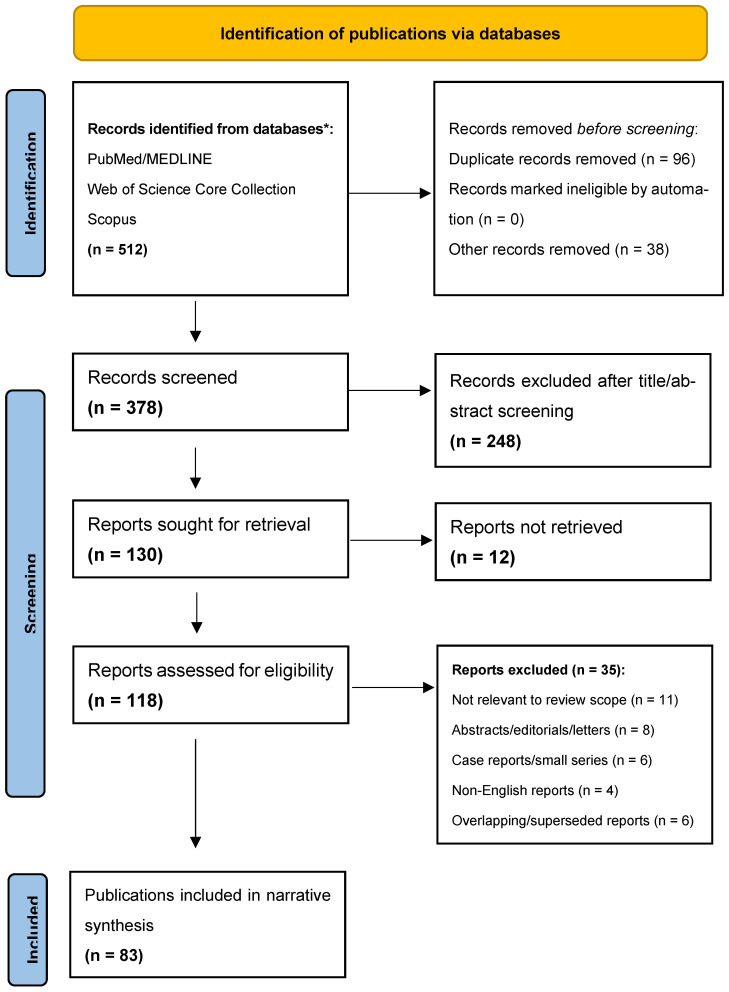
PRISMA-style flow diagram illustrating the identification, screening, eligibility assessment, and final selection of publications included in the narrative review. A total of 83 publications published between 2020 and 2026 were included in the narrative synthesis. One additional methodological reference concerning the SANRA recommendations was cited separately and was not included in the formal literature selection process. * The total number of records identified across PubMed/MEDLINE, Web of Science Core Collection, and Scopus is reported.

Titles and abstracts were independently screened by two authors, followed by full-text assessment of potentially eligible publications. Disagreements were resolved through discussion and consensus. The database search identified 512 records. After removal of 96 duplicates and 38 records for other predefined reasons, 378 records underwent title and abstract screening. Of these, 248 were excluded, and 130 reports were sought for retrieval. Twelve reports could not be retrieved, leaving 118 full-text reports for eligibility assessment. Thirty-five reports were excluded because they were not directly relevant to the review scope (n = 11), were conference abstracts, editorials, or letters (n = 8), were case reports or very small case series (n = 6), were published in languages other than English (n = 4), or represented overlapping or superseded reports (n = 6). A total of 83 publications were included in the final narrative synthesis. The detailed publication-selection process is presented in the PRISMA-style flow diagram in Figure 1. The 2019 SANRA publication was cited separately as a methodological reference and was not included in the formal selection process.

A domain-specific evidence hierarchy was applied. For clinical and procedural conclusions, the greatest weight was assigned to contemporary guidelines and expert consensus documents, randomized clinical trials and predefined trial substudies, systematic reviews and meta-analyses, and large prospective multicenter studies. Registries, observational studies, and intracoronary imaging cohorts were considered intermediate-level evidence. Histopathological, autopsy, translational, and mechanistic studies were treated as primary evidence for pathological definitions and biological interpretation, whereas narrative reviews and expert opinion were used mainly to provide context when higher-level evidence was unavailable.

Several methodological limitations should be acknowledged. Although a structured and reproducible search strategy was used, this study was a narrative review and was not based on a prospectively registered systematic review protocol. No formal risk-of-bias assessment or quantitative certainty-of-evidence framework was applied because the review integrated heterogeneous evidence types. The search was restricted to three databases, English-language publications, and the period from 2020 to 2026; therefore, relevant studies indexed elsewhere or published in other languages may have been omitted. Differences in calcified nodule terminology, imaging criteria, study populations, procedural techniques, and clinical endpoints limited direct comparisons among studies. Moreover, much of the lesion-specific evidence remains derived from observational studies, relatively small imaging cohorts, and expert consensus. Consequently, the proposed morphology-guided treatment framework should be regarded as an evidence-informed clinical synthesis rather than a formally validated therapeutic guideline.

## 3. Biological and Biomechanical Evolution of Coronary Calcification: From Early Mineralization to Calcified Nodules

Coronary calcification represents a dynamic but mechanistically heterogeneous continuum comprising two interrelated phases. The early phase is predominantly driven by metabolic, inflammatory, and osteogenic processes, including endothelial dysfunction, oxidative stress, extracellular vesicle-mediated mineralization, apoptosis, impaired efferocytosis, and osteogenic differentiation of vascular smooth muscle cells [10,34,35,36,37,38,39,40,41,42,43,44]. These mechanisms initiate hydroxyapatite nucleation and promote the progression from microscopic calcium deposits to spotty calcification and confluent sheet calcium.

The late phase is characterized primarily by biomechanical transformation of mature calcific plaques. Progressive calcium accumulation increases plaque stiffness and alters local stress distribution, particularly in tortuous coronary segments and regions exposed to repetitive bending, torsion, cyclic strain, and hinge motion [17,18,20,30,32]. These mechanical forces may cause fragmentation and fracture of mature calcific plates, followed by nodular protrusion, luminal surface disruption, thrombus formation, and the development of an eruptive calcified nodule. Distinguishing early metabolic mineralization from late biomechanical calcific plate failure provides a mechanistic framework for interpreting the heterogeneous pathological and imaging phenotypes of coronary calcium. This two-stage model is illustrated in Figure 1.

### 3.1. Coronary Calcification as an Active Biological Process

Coronary artery calcification is increasingly recognized as a dynamic and highly regulated biological process rather than a passive consequence of aging and plaque degeneration. Similar to physiological bone formation, vascular calcification is governed by complex interactions among chronic inflammation, oxidative stress, endothelial dysfunction, lipid accumulation, and disturbances in mineral metabolism [8,11,34,35,36]. These mechanisms collectively promote the deposition of calcium-phosphate crystals within the vascular wall and contribute to progressive plaque remodeling.

A key event in the initiation of vascular calcification is the phenotypic transformation of vascular smooth muscle cells (VSMCs). Under physiological conditions, VSMCs maintain vascular tone and structural integrity through a contractile phenotype. Exposure to inflammatory cytokines, oxidized lipoproteins, hyperglycemia, and oxidative stress induces an osteogenic transition in VSMCs. These cells begin to express bone-related transcription factors and proteins, including runt-related transcription factor 2 (RUNX2), bone morphogenetic proteins (BMPs), osteocalcin, osteopontin, and alkaline phosphatase [8,9,35,36]. This osteogenic reprogramming promotes extracellular matrix mineralization and progressive calcium accumulation within atherosclerotic plaques.

Inflammation plays a central role in vascular mineralization. Activated endothelial cells recruit monocytes and macrophages, which release pro-inflammatory mediators such as tumor necrosis factor-α (TNF-α), interleukin-1β (IL-1β), and interleukin-6 (IL-6) [8,9,34,35,36,37]. These cytokines stimulate osteogenic signaling and accelerate vascular mineralization. Activation of the NLRP3 inflammasome further links chronic inflammation with cellular injury and calcification [38]. In parallel, reactive oxygen species promote mitochondrial dysfunction, DNA damage, and cellular senescence, thereby sustaining a self-perpetuating pro-calcific cycle [8,9,11,34,35,36].

Recent evidence suggests that apoptosis of vascular smooth muscle cells and defective clearance of apoptotic debris by macrophages (impaired efferocytosis) represent additional drivers of vascular mineralization. The accumulation of apoptotic bodies within the necrotic core provides a favorable microenvironment for calcium-phosphate crystal nucleation and contributes to the development of early calcific deposits [39]. These processes are particularly relevant in advanced atherosclerotic plaques, where persistent inflammation and impaired tissue repair accelerate plaque calcification.

The mechanisms driving vascular calcification are especially pronounced in patients with diabetes mellitus, chronic kidney disease, and advanced coronary artery disease. Disturbances in phosphate metabolism, altered fibroblast growth factor-23 (FGF-23)/Klotho signaling, chronic low-grade inflammation, and metabolic stress promote osteogenic differentiation and accelerate mineral deposition within the vascular wall [40,41,42]. Consequently, these populations exhibit a disproportionate burden of severe coronary calcification and are more likely to develop complex calcific lesion phenotypes.

Collectively, these pathways establish the biological basis for early microcalcification, spotty calcification, and the subsequent formation of confluent sheet calcium [8,10,34,35,36].

### 3.2. The Role of Extracellular Vesicles and Microcalcification Formation

Recent evidence suggests that extracellular vesicles play a pivotal role in the initiation of vascular calcification. These membrane-bound particles are released by vascular smooth muscle cells, macrophages, and apoptotic cells in response to oxidative stress and inflammatory stimulation [10,43]. Extracellular vesicles contain phosphatidylserine, annexins, calcium-binding proteins, and mineralization-promoting enzymes that facilitate the nucleation of hydroxyapatite crystals and serve as the earliest foci of calcium deposition within the arterial wall [10,39,43,44].

Macrophage-derived extracellular vesicles appear to be particularly important in advanced atherosclerotic lesions. Through the release of pro-calcific microvesicles and inflammatory mediators, activated macrophages promote both mineralization and plaque progression. Simultaneously, defective efferocytosis enhances the accumulation of apoptotic debris and further increases the availability of nucleation sites for calcium crystal formation [44].

The aggregation of extracellular vesicles and apoptotic bodies leads to the formation of microcalcifications, typically defined as calcific deposits measuring less than 50 μm in diameter. Despite their small size, these deposits may markedly affect plaque biomechanics when located within or immediately adjacent to a thin fibrous cap. Calcium is substantially stiffer than the surrounding collagen-rich tissue. The interface between these materials therefore creates a mechanical discontinuity at which circumferential tensile stress becomes concentrated.

Stress amplification depends on microcalcification size, shape, orientation, proximity to the luminal surface, and spatial relationship with adjacent deposits. Closely spaced or clustered microcalcifications may generate interacting stress fields within an already attenuated fibrous cap. This interaction increases the likelihood of microscopic tissue failure and subsequent cap rupture [12,15,16,45].

Clinically, early or limited calcification should not invariably be interpreted as a marker of plaque stabilization. Broad confluent sheet calcium may be associated with relative plaque stability. In contrast, dispersed sub-50 μm microcalcifications within a thin fibrous cap may promote rupture, thrombosis, and acute coronary syndromes. Their clinical relevance therefore depends on size, distribution, and anatomical location rather than on total calcium burden alone [12,15,16,45].

This mechanism differs from the late-stage biomechanical fracture of mature calcific plates that promotes nodular protrusion and calcified nodule formation [12,15,17,18,30,32].

Microcalcifications represent the earliest stage of coronary mineralization. Continued inflammation, apoptosis, extracellular matrix remodeling, and mineral deposition promote their coalescence into spotty calcification and subsequently into confluent sheet calcium [18,20]. These mature calcific plates provide the structural substrate for later biomechanical fracture [17,18,20,30,32].

### 3.3. Late Biomechanical Transformation: From Sheet Calcium to Calcified Nodules

As microcalcifications enlarge and coalesce, they form deposits that become detectable by intravascular imaging. Early macroscopic mineralization frequently appears as spotty calcification, which is associated with persistent inflammation, macrophage infiltration, plaque progression, and adverse cardiovascular events [46,47]. Continued mineral deposition and extracellular matrix remodeling lead to the formation of confluent sheet calcium. Compared with spotty calcification, sheet calcium is generally less inflammatory and may contribute to plaque stability. However, it also increases vessel stiffness, reduces vascular compliance, and alters local stress distribution [13,18,20,34].

The transition from sheet calcium to nodular calcium and calcified nodules represents a late biomechanical stage rather than a continuation of metabolic mineral deposition. Broad, rigid calcific plates alter plaque mechanics and concentrate stress at the interface with the more compliant surrounding tissue. In tortuous or highly mobile coronary segments, repetitive bending, torsion, hinge motion, and pulsatile loading may fracture these plates. The resulting fragments may then protrude into the vessel lumen [17,18,20,30,32].

This process has led to an important distinction between nodular calcium and calcified nodules. Nodular calcium refers to a protruding calcific mass without clear evidence of luminal surface disruption or superimposed thrombosis. In contrast, a calcified nodule is characterized by fractured calcific plates, irregular or eruptive calcium protruding into the lumen, surface disruption, and, in selected lesions, thrombus formation [13,17,30,31,32,48,49].

Histopathological observations support the concept that repetitive mechanical loading fractures mature calcific plates and promotes luminal protrusion of calcific fragments, particularly in coronary segments exposed to marked vessel motion [13,17,18,20].

Interestingly, calcified nodules are not randomly distributed throughout the coronary tree. Pathological and imaging studies have demonstrated a predilection for the right coronary artery and segments exposed to increased mechanical stress, vessel tortuosity, or hinge motion [48,50]. These observations further support the concept that biomechanical factors play a central role in calcified nodule formation and progression.

Importantly, contemporary intravascular imaging studies suggest that not all calcified nodules are associated with acute thrombosis. Not all imaging-defined calcified nodules are associated with acute thrombosis. Many lesions identified by OCT or IVUS appear clinically stable and may represent intermediate phenotypes between nodular calcium and an eruptive calcified nodule [49,51]. These findings broaden the traditional pathological concept of calcified nodules and support their classification as a heterogeneous spectrum of lesions.

**Figure 2:**
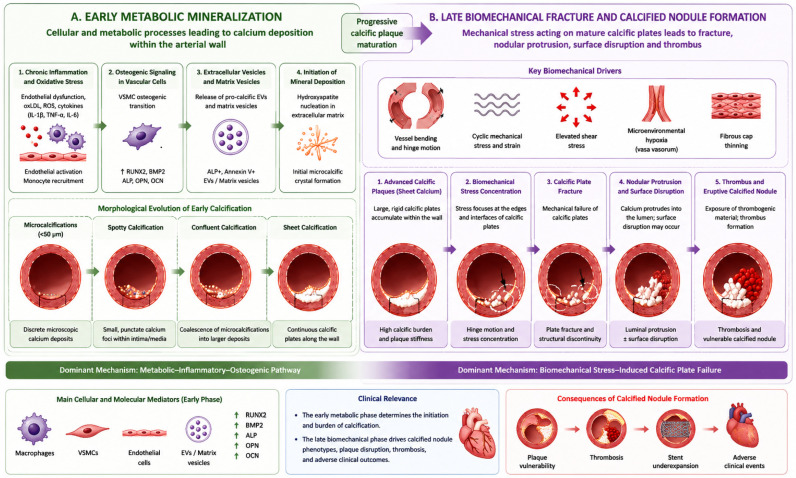
Consolidated Mechanistic Pathway Linking Early Metabolic Mineralization to Late Biomechanical Calcific Plate Fracture and Calcified Nodule Formation. Panel (**A**) depicts the early metabolic, inflammatory, and osteogenic phase of coronary calcification. Endothelial dysfunction, oxidative stress, macrophage activation, vascular smooth muscle cell osteogenic differentiation, apoptosis, impaired efferocytosis, and extracellular vesicle release promote hydroxyapatite nucleation and the progression from sub-50 μm microcalcifications to spotty calcification and confluent sheet calcium. Microcalcifications located within or adjacent to a thin fibrous cap may create focal stress concentrations and increase cap vulnerability. Panel (**B**) depicts the late biomechanical phase, in which mature calcific plates exposed to repetitive vessel bending, hinge motion, torsion, cyclic strain, and shear stress undergo fragmentation and structural fracture, resulting in nodular calcium, luminal protrusion, surface disruption, thrombus formation, and an eruptive calcified nodule. The figure emphasizes that early mineralization is predominantly driven by cellular and metabolic mechanisms, whereas calcified nodule formation is primarily associated with biomechanical failure of advanced calcific plates. The figure represents an author-developed synthesis of mechanistic, biomechanical, and histopathological evidence [8,9,10,11,12,13,14,15,16,17,18,20,30,32,34,35,36,37,38,39,40,41,42,43,44,45,46,47]. Abbreviations: ALP, alkaline phosphatase; BMP2, bone morphogenetic protein 2; EV, extracellular vesicle; IL, interleukin; OCN, osteocalcin; OPN, osteopontin; oxLDL, oxidized low-density lipoprotein; ROS, reactive oxygen species; RUNX2, runt-related transcription factor 2; TNF-α, tumor necrosis factor-α; VSMC, vascular smooth muscle cell.

Collectively, coronary calcification follows a two-stage mechanistic model. Early metabolic, inflammatory, and osteogenic processes promote the progression from microcalcifications to spotty calcification and confluent sheet calcium, whereas late biomechanical loading may fracture mature calcific plates and generate nodular or eruptive calcified phenotypes [17,18,20,30,32,48,49,50,51]. This distinction is summarized in Figure 2.

## 4. Pathobiology and Clinical Significance of Calcified Nodules

Calcified nodules are clinically relevant fibrocalcific lesions with heterogeneous morphology, thrombotic potential, and procedural consequences. This section reviews their pathological definition, anatomical distribution, clinical presentation, and impact on PCI outcomes [17,18,49,50,51].

### 4.1. Calcified Nodules as a Distinct Coronary Plaque Phenotype

Calcified nodules represent a distinct and advanced phenotype of fibrocalcific coronary atherosclerosis characterized by disrupted calcific plates and protruding nodular calcium extending into the vessel lumen, with or without associated thrombosis [13,17,48,49]. Originally described by Virmani and colleagues as one of the pathological substrates of coronary thrombosis, calcified nodules differ fundamentally from plaque rupture and plaque erosion with respect to both morphology and pathogenesis [17]. Whereas plaque rupture is characterized by fibrous cap disruption overlying a lipid-rich necrotic core and plaque erosion typically involves endothelial denudation without cap rupture, calcified nodules arise primarily from the mechanical fragmentation of dense fibrocalcific plaques [17,18,30,48].

The pathological hallmark of calcified nodules is eruptive calcium penetrating the luminal surface. Histological studies have demonstrated fractured calcific plates, irregular luminal architecture, fibrin deposition, and variable degrees of superimposed thrombosis [13]. Contemporary imaging studies suggest, however, that calcified nodules encompass a broader spectrum of lesions than originally described in pathological series. Many lesions identified by OCT exhibit protruding nodular calcium without overt thrombus formation, indicating that imaging-defined calcified nodules may represent different stages within a continuum of calcific plaque evolution [32,49]. Recent lesion-specific and systematic reviews further emphasize that eruptive and non-eruptive calcified nodules represent distinct morphological phenotypes with different thrombotic potential and procedural implications for PCI [31,32].

Calcified nodules are particularly prevalent in elderly individuals and in patients with diabetes mellitus, chronic kidney disease, or extensive coronary calcification [18,34]. These conditions accelerate vascular mineralization and increase susceptibility to calcium fragmentation through both biological and biomechanical mechanisms. Consequently, calcified nodules are frequently encountered in heavily calcified vessels with a substantial overall plaque burden and may represent one of the most advanced manifestations of fibrocalcific coronary artery disease [18,34].

### 4.2. Epidemiology and Anatomical Distribution

The true prevalence of calcified nodules remains incompletely understood and varies considerably according to the diagnostic modality and study population. Early autopsy studies suggested that calcified nodules account for approximately 2–7% of thrombotic coronary lesions associated with sudden cardiac death [17]. However, the widespread adoption of OCT and IVUS has revealed a substantially higher prevalence of calcified nodule-like lesions, particularly among patients undergoing PCI for severe coronary calcification [49].

Several studies have demonstrated a predilection of calcified nodules for the right coronary artery (RCA), especially within segments exposed to pronounced vessel curvature, hinge motion, and repetitive mechanical stress [17,18,30,32,49]. Similar lesions may occur in the left anterior descending and left circumflex arteries but appear less frequently than in the RCA. This characteristic anatomical distribution provides additional support for the role of biomechanical stress in calcified nodule formation [17,18,30,32,49].

In addition to anatomical factors, several clinical conditions appear to predispose patients to calcified nodule development. Advanced age, diabetes mellitus, chronic kidney disease, dialysis dependence, and diffuse coronary calcification have consistently been associated with a higher prevalence of these lesions [18,29,32]. Collectively, these observations suggest that calcified nodules represent a final common pathway resulting from the interaction of chronic vascular mineralization and repetitive mechanical stress [18,34,50].

### 4.3. Calcified Nodules and Acute Coronary Syndromes

Although calcified nodules account for a smaller proportion of acute coronary syndromes than plaque rupture or plaque erosion, they nevertheless represent a distinct mechanism of coronary thrombosis [48]. Protrusion of fractured calcium into the vessel lumen may disrupt endothelial integrity, expose thrombogenic material, and generate regions of disturbed flow that facilitate platelet activation and thrombus formation [17,48].

Histopathological studies have demonstrated fibrin-rich thrombi overlying eruptive calcified nodules, supporting their role as potential triggers of acute coronary events [17]. Nevertheless, contemporary intracoronary imaging studies indicate that many calcified nodules identified in vivo remain clinically stable and are not associated with acute thrombosis [30,31,32,49,51]. OCT studies have further demonstrated that protruding nodular calcium is frequently encountered in stable coronary artery disease, suggesting that thrombogenicity may depend on lesion-specific morphological characteristics rather than calcium protrusion alone [34,49,51].

This discrepancy likely reflects differences between pathological definitions and imaging-based classifications and suggests that calcified nodules encompass a heterogeneous spectrum of lesions with variable thrombotic potential [17,48,51]. Consequently, the clinical significance of a calcified nodule may depend on multiple factors, including the degree of luminal protrusion, presence of endothelial disruption, thrombus burden, and overall plaque morphology. Future studies are needed to determine which imaging features identify lesions at highest risk for future coronary events and whether specific lesion subsets require more aggressive treatment strategies [34,49,51].

### 4.4. Calcified Nodules and Adverse PCI Outcomes

Beyond their association with acute coronary syndromes, calcified nodules have emerged as an important determinant of PCI complexity and long-term procedural outcomes. The protruding and irregular morphology of these lesions may impair balloon expansion, hinder optimal stent deployment, and increase the likelihood of residual stent underexpansion [19,22]. Among these mechanisms, stent underexpansion is considered one of the strongest predictors of adverse outcomes in heavily calcified lesions and likely represents the principal pathway linking calcified nodules with target lesion failure [19].

Several OCT and IVUS studies, together with recent lesion-specific and systematic reviews, have demonstrated that nodular calcium and calcified nodules are associated with suboptimal stent expansion, malapposition, restenosis, target lesion revascularization, and recurrent ischemic events [31,32,49,50]. Furthermore, these lesions frequently require advanced calcium-modification strategies, including RA, OA, ELCA, or IVL before definitive stent implantation [3].

The presence of calcified nodules may also influence procedural decision-making by necessitating more aggressive lesion preparation and greater reliance on intravascular imaging for optimization of PCI results [3,22]. Consequently, accurate identification and characterization of calcified nodules have become central objectives of contemporary intravascular imaging, underscoring the need for standardized imaging criteria and lesion-specific treatment strategies [3,22].

Collectively, these findings indicate that calcified nodules should be regarded not merely as a pathological curiosity but as clinically relevant lesions with important implications for coronary thrombosis, procedural success, and long-term PCI outcomes [3,5,31,52]. Their recognition has become increasingly important in the era of precision PCI, where treatment strategies are progressively tailored according to detailed lesion morphology and intravascular imaging findings [3,22].

The key pathological, imaging, clinical, and procedural differences between sheet calcium, nodular calcium, and calcified nodules are summarized in Table 1.

## 5. Multimodality Intravascular Imaging of Coronary Calcification and Calcified Nodules

Intravascular imaging is essential for distinguishing calcific phenotypes because conventional angiography provides limited information on calcium morphology and distribution. OCT and IVUS enable detailed assessment of calcium architecture, plaque burden, vessel dimensions, and lesion vulnerability [22,25,52,53,54]. NIRS provides complementary information on plaque composition. Together, these modalities support procedural planning, calcium-modification device selection, and PCI optimization [3,22,25,49,54]. Recent randomized evidence further supports this approach. The CALIPSO trial demonstrated improved stent implantation results with algorithm-based OCT guidance compared with angiographic guidance in calcified coronary lesions, while the 5-year results of RENOVATE-COMPLEX-PCI showed improved long-term clinical outcomes with intravascular imaging-guided complex PCI [55,56].

### 5.1. Limitations of Coronary Angiography in the Assessment of Calcified Lesions

Coronary angiography remains the cornerstone of diagnostic and interventional cardiology; however, its ability to characterize coronary calcification is limited. Angiographic calcium is typically identified as radiopaque densities visible before contrast injection and frequently appears as linear or irregular opacities within the vessel wall [52]. Severe calcification can often be recognized angiographically. However, angiography provides only a two-dimensional lumenogram and cannot reliably assess calcium thickness, depth, circumferential distribution, plaque composition, or specific calcific phenotypes [52,53].

Several studies have demonstrated that angiography substantially underestimates the extent and severity of coronary calcification compared with intravascular imaging modalities [23,53]. Moreover, angiography cannot reliably distinguish among different calcific phenotypes, including sheet calcium, nodular calcium, and calcified nodules. As a result, lesion morphology and procedural complexity may be underestimated, potentially leading to inadequate lesion preparation, inappropriate device selection, and suboptimal PCI outcomes [3,22,53].

Consequently, contemporary interventional practice increasingly relies on intravascular imaging for comprehensive characterization of calcified lesions. Current expert consensus documents recommend the use of IVUS or OCT whenever detailed assessment of calcium morphology is expected to influence procedural planning, calcium-modification strategy selection, or stent optimization [3,22,54].

### 5.2. Intravascular Ultrasound (IVUS)

Intravascular ultrasound (IVUS) is one of the most widely utilized imaging modalities for the assessment of calcified coronary lesions. Owing to its excellent tissue penetration, IVUS enables visualization of both superficial and deep calcium and provides comprehensive information regarding plaque burden, vessel dimensions, lesion length, and overall coronary architecture [22,23,25,52].

On IVUS, calcium appears as a hyperechoic structure with acoustic shadowing [22,23,52]. Although shadowing prevents reliable measurement of calcium thickness, IVUS allows assessment of calcium arc, longitudinal extent, and overall burden [22,23,52,57]. IVUS-derived scoring systems can help predict stent underexpansion and guide lesion preparation. High-risk features include a calcium arc >270°, calcium length >5 mm, circumferential superficial calcium, and a calcified nodule [3,23,52].

Calcified nodules exhibit characteristic IVUS features. On IVUS, calcified nodules appear as convex calcium protrusions with an irregular luminal surface and acoustic shadowing. Disruption of the underlying calcific sheet may occasionally be visible. However, the limited spatial resolution of IVUS makes differentiation between nodular calcium and a true eruptive calcified nodule challenging [30,31,49,52].

Beyond lesion characterization, IVUS plays an important role in procedural planning and optimization. Accurate assessment of vessel dimensions, plaque burden, and calcium distribution facilitates selection of appropriate calcium-modification devices and contributes to improved stent sizing and expansion [3,22,23,25]. In heavily calcified lesions, IVUS guidance has been associated with improved procedural outcomes and reduced rates of stent failure compared with angiography-guided PCI alone [21,22,55].

The major advantages of IVUS include deep tissue penetration, reliable assessment of plaque burden, and accurate vessel sizing. Furthermore, IVUS provides superior visualization of deep calcium deposits that may not be fully appreciated with OCT. However, its lower spatial resolution compared with OCT limits visualization of fibrous cap integrity, superficial calcium microstructure, and small thrombi associated with calcified nodules [22,25,56,58].

### 5.3. Optical Coherence Tomography (OCT)

OCT provides the highest spatial resolution among currently available intracoronary imaging modalities and is therefore the reference technique for detailed assessment of superficial coronary calcium morphology. It uses near-infrared light to generate cross-sectional images of superficial plaque structures with near-histological resolution [24,53,59]. The 2025 expert consensus update provides contemporary standards for quantitative OCT measurements and the morphological assessment of coronary lesions [53].

On OCT, calcium appears as a signal-poor region with sharply delineated borders, enabling direct measurement of calcium thickness, arc, and longitudinal extension [24,53,59]. These characteristics have facilitated the development of OCT-based calcium scoring systems that predict stent expansion and identify lesions likely to require calcium-modification therapies. The widely used OCT calcium score incorporates maximum calcium arc, thickness, and length. Higher scores predict an increased risk of stent underexpansion and procedural failure [19].

OCT is particularly valuable for identifying calcified nodules. Consensus definitions describe these lesions as calcium masses protruding into the vessel lumen with an irregular luminal surface and sharply delineated borders [30,31,49,53]. Supportive features include fibrous cap disruption, superficial calcium fracture, thrombus, and eruptive calcium arising from an underlying calcific plate [48,49]. Owing to its high spatial resolution, OCT can distinguish among sheet calcium, nodular calcium, and calcified nodules, making it the preferred modality for morphological classification of calcific lesions [24,30,31,49,53,59].

OCT can also identify associated features such as fibrous cap disruption, superficial calcium fractures, macrophage accumulation, microthrombi, and luminal irregularities [44]. These findings may provide additional information on lesion vulnerability and thrombotic potential. However, protruding nodular calcium is also frequently observed in stable coronary artery disease, indicating that calcium protrusion alone should not be considered evidence of thrombogenicity [51]. This observation supports the concept that calcified nodules represent a heterogeneous spectrum of lesions with variable clinical significance [51].

In addition to lesion characterization, OCT has become an important tool for procedural guidance. Precise measurements of calcium arc, thickness, and length allow operators to predict the likelihood of stent underexpansion and determine the need for atherectomy or intravascular lithotripsy [19]. OCT can also identify calcium fractures after lesion preparation, providing direct evidence of procedural effectiveness before stent implantation [26,27,60]. The CALIPSO randomized trial further supports the use of a predefined OCT-guided strategy for lesion preparation, stent sizing, and procedural optimization in calcified coronary lesions [56].

Despite these advantages, OCT is limited by relatively shallow tissue penetration and reduced visualization of deep calcium deposits [22,25,58]. OCT and IVUS should therefore be regarded as complementary rather than competing imaging modalities. OCT is superior for evaluating superficial calcium morphology and calcified nodules, whereas IVUS provides better assessment of overall plaque burden, vessel dimensions, and deep calcium distribution [22,25,54,58].

### 5.4. Near-Infrared Spectroscopy and Hybrid NIRS-IVUS Imaging

Near-infrared spectroscopy (NIRS) has emerged as an important adjunctive intravascular imaging modality capable of identifying lipid-rich coronary plaques. Unlike IVUS and OCT, NIRS does not directly visualize calcium morphology or plaque architecture. Instead, it analyzes the spectral characteristics of the vessel wall and identifies lipid-core plaques that may contribute to plaque vulnerability and adverse cardiovascular outcomes [61].

The introduction of hybrid NIRS-IVUS systems has enabled simultaneous assessment of plaque composition and vessel morphology. This combined approach provides complementary information by integrating the structural assessment of IVUS with the compositional analysis offered by NIRS [61,62,63,64]. Consequently, NIRS-IVUS allows comprehensive evaluation of plaque burden, calcification, vessel dimensions, and lipid content within a single imaging acquisition [62].

NIRS findings are typically expressed using the lipid core burden index (LCBI), which quantifies the amount of lipid-rich plaque within the examined vessel segment. The maximum lipid core burden index within a 4-mm segment (maxLCBI4mm) is the most commonly used NIRS parameter. Elevated maxLCBI4mm values have been associated with vulnerable plaques, future coronary events, distal embolization, slow-flow phenomena, and periprocedural myocardial infarction during PCI [61,62,63].

NIRS cannot directly diagnose calcified nodules. However, lipid-rich plaque may coexist with severe calcification in advanced lesions [61,64]. Hybrid NIRS-IVUS can therefore identify lesions that combine a high calcium burden with lipid-rich plaque. Such lesions may be associated with both procedural complexity and increased thrombotic potential [61,64,65,66,67].

Recent studies have demonstrated that lipid-rich plaques adjacent to heavily calcified segments may be associated with an increased risk of procedural complications, including distal embolization, slow-flow phenomena, and periprocedural myocardial injury [62,63]. In this context, NIRS-IVUS may provide incremental value by identifying patients who could benefit from intensified procedural planning, embolic protection strategies, or more aggressive secondary prevention [63,67].

Another advantage of hybrid NIRS-IVUS imaging is its ability to improve risk stratification beyond anatomical assessment alone. While IVUS provides detailed information regarding calcium burden and vessel geometry, NIRS offers complementary insights into plaque biology. The integration of these modalities aligns with the broader concept of precision PCI, in which treatment strategies are increasingly tailored according to both lesion morphology and plaque composition [3,22].

The role of NIRS in calcified nodules remains incompletely defined. NIRS should be regarded as a complementary tool for assessing plaque composition rather than as a direct diagnostic modality. When combined with IVUS, it may improve characterization of complex calcified lesions and support procedural planning and long-term risk assessment [3,22,61,62,63,64,65].

**Figure 3:**
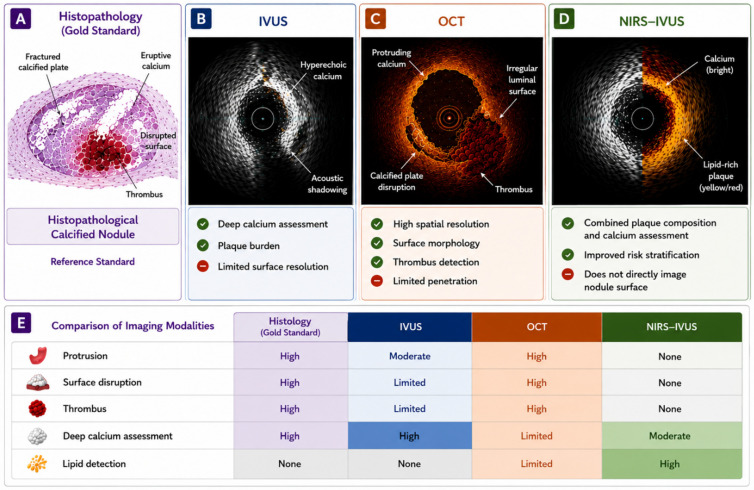
Complementary Roles of IVUS, OCT, and NIRS-IVUS in the Assessment of Calcified Nodules and Associated Plaque Characteristics. IVUS provides deep tissue penetration and enables assessment of vessel dimensions, plaque burden, calcium arc, longitudinal calcium extent, deep calcium distribution, and acoustic shadowing; however, it does not permit reliable measurement of calcium thickness and has limited ability to identify superficial cap disruption or small thrombi. OCT provides high-resolution characterization of superficial calcium morphology and enables direct measurement of calcium arc, thickness, and length, as well as visualization of luminal protrusion, surface disruption, thrombus, and calcium fractures; its principal limitation is reduced penetration into deep plaque structures. NIRS-IVUS combines the structural information provided by IVUS with the detection and quantification of lipid-rich plaque using the lipid core burden index, but it does not directly identify calcified nodules. Together, these modalities provide complementary structural, morphological, and compositional information that may improve lesion classification, procedural planning, and selection of calcium-modification strategies. The figure represents an author-developed synthesis based on intravascular imaging studies and consensus definitions [22,23,24,25,30,31,49,52,53,59,61,62,63,64,65]. Abbreviations: IVUS, intravascular ultrasound; LCBI, lipid core burden index; maxLCBI4mm, maximum lipid core burden index within a 4-mm segment; NIRS, near-infrared spectroscopy; OCT, optical coherence tomography.

The complementary strengths and limitations of contemporary intravascular imaging modalities in the assessment of calcified nodules and associated plaque characteristics are summarized in Figure 3.

As illustrated in Figure 3, no single modality provides a complete assessment of calcified nodules. Combining structural and compositional imaging information may improve lesion classification and inform the selection of calcium-modification strategies [3,22,49].

### 5.5. Diagnostic Criteria for Calcified Nodules Across IVUS, OCT, and NIRS

Accurate identification of calcified nodules remains challenging because definitions vary among pathological studies, intravascular imaging modalities, and clinical investigations. As a result, considerable heterogeneity exists in the reported prevalence, morphology, and clinical significance of calcified nodules [18,49].

Pathologically, calcified nodules are characterized by fractured calcific plates, eruptive calcium protruding into the lumen, surface disruption, and frequently superimposed thrombus [13,17]. Many of these features cannot be fully resolved using contemporary intravascular imaging. Consequently, pathological and imaging-based definitions are not completely concordant [18,49].

OCT currently provides the most detailed assessment of calcified nodule morphology owing to its superior spatial resolution [24,53,59]. Consensus definitions describe calcified nodules as calcium masses that protrude into the lumen and have an irregular surface with sharply delineated borders [30,31,49,53]. Supportive features include superficial calcium fracture, fibrous cap disruption, luminal irregularity, and thrombus [17,30,31,48,49,53]. Because OCT directly visualizes these superficial structures, it is the reference intravascular modality for calcified nodule characterization [24,30,31,49,53,59].

IVUS identifies calcified nodules as convex luminal calcium protrusions with an irregular surface and acoustic shadowing [30,31,49,52]. Disruption of the underlying calcific plate may occasionally be visible [49,55]. IVUS provides excellent assessment of overall plaque burden and deep calcium distribution. However, its lower spatial resolution limits visualization of superficial microstructure, thrombus, and fibrous cap integrity. Differentiation between nodular calcium and a true calcified nodule is therefore more challenging with IVUS than with OCT [22,24,51,52,59,68].

In contrast, NIRS does not directly identify calcified nodules. Rather, it provides complementary information regarding plaque lipid content and biological vulnerability [61,62,63,64,65]. Therefore, NIRS should not be considered a diagnostic modality for calcified nodules but may contribute to characterization of the surrounding plaque environment and overall lesion risk profile [3,61,66].

Importantly, contemporary imaging studies have demonstrated that imaging-defined calcified nodules are substantially more common than pathological calcified nodules identified in autopsy series [17,18,49]. This discrepancy suggests that protruding nodular calcium visualized by OCT or IVUS may represent a broader spectrum of lesions, ranging from relatively stable nodular calcium to fully developed eruptive calcified nodules with thrombotic potential [30,31,32,49]. Consequently, the terms nodular calcium and calcified nodule should not be used interchangeably, particularly when interpreting imaging studies and comparing results across different investigations [49,51].

Collectively, these observations highlight the complementary nature of contemporary intravascular imaging modalities. The three modalities provide complementary information. OCT is preferred for detailed assessment of superficial calcified nodule morphology. IVUS provides superior evaluation of vessel architecture and deep calcium burden, whereas NIRS characterizes plaque composition [22,25,57,61,62,63,64,65]. Their integration may support more comprehensive lesion assessment and individualized treatment planning [3,22,49]. Future efforts should focus on the development of standardized imaging definitions and multimodality diagnostic algorithms to improve consistency across clinical studies and routine practice [30,31,32,53].

### 5.6. Lack of Standardized Diagnostic Criteria: Implications for Research and Clinical Practice

The absence of standardized criteria for calcified nodules limits both clinical research and routine practice [30,31,32,53]. Pathological definitions usually require fractured calcific plates, eruptive calcium, luminal surface disruption, and often superimposed thrombus [17,18]. Imaging studies may classify convex or irregular calcific protrusions as calcified nodules even when fracture, surface disruption, or thrombus cannot be demonstrated [30,31,32,49,51,52,53]. Consequently, the same term may describe biologically distinct lesions, ranging from relatively stable nodular calcium to thrombogenic eruptive calcified nodules.

This definitional heterogeneity has a direct impact on reported prevalence. Studies applying broad OCT- or IVUS-based criteria may identify more calcified nodules than pathological series or imaging studies requiring evidence of surface disruption, calcium eruption, or superimposed thrombus [17,18,32]. Reported prevalence depends on the study population, imaging modality, spatial resolution, diagnostic threshold, culprit-lesion status, and terminology used [30,31,32,53]. Comparisons among studies should therefore be interpreted cautiously, particularly when different nodular phenotypes are analyzed as a single category. Comparisons among studies should consequently be interpreted with caution, particularly when nodular calcium, non-eruptive calcified nodules, and eruptive calcified nodules are analysed as a single lesion category.

Definitional heterogeneity may also explain inconsistent prognostic findings. Combining stable calcific protrusions with disrupted or thrombotic nodules may weaken associations with adverse outcomes [30,31,32,49,50]. Conversely, studies restricted to culprit or eruptive nodules may overestimate the risk associated with imaging-defined calcified nodules as a whole. OCT-based analyses support separate reporting of eruptive and non-eruptive morphologies [49]. Future studies should report surface disruption, thrombus, luminal protrusion, calcium plate fracture, and culprit-lesion status.

Diagnostic uncertainty may influence treatment selection. Labeling a smooth calcific protrusion as a calcified nodule may lead to unnecessarily aggressive lesion preparation. Conversely, failure to recognize a markedly protruding or eruptive nodule may result in inadequate modification and residual stent underexpansion [19,30,31,49,54]. No single calcium-modification strategy is appropriate for all imaging-defined calcified nodules [30,31,54]. Device selection should therefore be based on lesion morphology, calcium depth and distribution, vessel dimensions, balloon deliverability, and thrombus burden rather than on the diagnostic label alone [3,5,30,31,54].

To improve comparability and clinical translation, future studies should adopt modality-specific but harmonized definitions. Future studies should adopt harmonized, modality-specific definitions. Investigators should distinguish sheet calcium, nodular calcium, non-eruptive calcified nodules, and eruptive calcified nodules [30,31,32,53]. They should also report the imaging modality, diagnostic criteria, culprit status, surface disruption, thrombus, calcium arc, thickness, length, depth, and degree of luminal protrusion. Independent core-laboratory adjudication and prospective validation against histopathological or multimodality imaging standards would further improve reproducibility [30,31,32,49,53]. Until such criteria are established, calcified nodules should be described according to their individual morphological components rather than treated as a uniform binary diagnosis.

## 6. Imaging-Guided Calcium Modification and Precision PCI Strategies

The increasing prevalence of severe coronary calcification has stimulated the development of multiple calcium-modification technologies designed to facilitate PCI in complex lesions [3,5,54,67]. Contemporary treatment strategies increasingly rely on intravascular imaging to guide device selection, lesion preparation, and stent optimization [3,22,53,55,56]. RA, OA, ELCA, IVL, and hybrid approaches target different components of calcium morphology and vessel compliance. Recent consensus documents and randomized trials have provided important new evidence regarding the selection and limitations of these strategies [69,70,71,72]. Consequently, modern PCI is evolving toward a morphology-guided approach in which treatment is tailored to intravascular imaging findings rather than angiographic appearance alone.

### 6.1. Why Calcified Nodules Require a Dedicated PCI Approach

Calcified nodules represent one of the most challenging lesion subsets encountered during contemporary PCI. Unlike concentric sheet calcium, calcified nodules are characterized by irregular luminal protrusion, fragmented calcific architecture, and frequently heterogeneous plaque composition. These features may impair guidewire crossing, reduce balloon expansion, hinder stent delivery, and increase the risk of residual stent underexpansion [19,22,73].

Among all procedural complications associated with heavily calcified lesions, stent underexpansion remains one of the strongest predictors of adverse clinical outcomes. Inadequate stent expansion has been consistently associated with in-stent restenosis, stent thrombosis, target lesion revascularization, and recurrent ischemic events [19,67,73]. Because calcified nodules frequently create focal areas of extreme mechanical resistance, they often require more aggressive lesion preparation than conventional calcified plaques [30,31,49,54].

The increasing availability of intravascular imaging has fundamentally changed the management of these lesions. Rather than relying solely on angiographic appearance, operators can now assess calcium arc, thickness, depth, length, luminal protrusion, and the presence of calcified nodules before selecting an appropriate calcium-modification strategy [19,22,54]. Consequently, contemporary treatment algorithms increasingly emphasize imaging-guided decision-making as a cornerstone of precision PCI [3,22].

Importantly, the optimal calcium-modification strategy depends not only on calcium severity but also on lesion morphology. Superficial concentric calcium, deep sheet calcium, protruding nodular calcium, and eruptive calcified nodules may require different treatment approaches, highlighting the importance of pre-procedural IVUS or OCT assessment [3,22,54]. In particular, OCT-derived measurements of calcium arc, thickness, and length have emerged as important determinants of lesion compliance and procedural strategy selection [19,59].

### 6.2. Balloon-Based Calcium Modification

Specialized balloon technologies remain the most commonly used initial treatment option for calcified coronary lesions. These devices aim to modify calcium architecture, improve vessel compliance, and facilitate optimal stent expansion [3,5,74,75].

#### 6.2.1. Non-Compliant Balloons

Non-compliant (NC) balloons remain the foundation of lesion preparation. Owing to their limited expansion beyond the nominal diameter, NC balloons generate high focal pressure and may successfully dilate mild-to-moderate calcified lesions. However, their effectiveness is often limited in severe calcification and calcified nodules, where rigid calcium deposits resist balloon expansion [74,75].

#### 6.2.2. Cutting and Scoring Balloons

Cutting and scoring balloons were developed to improve plaque modification while reducing uncontrolled vessel injury. These devices create controlled plaque incisions and facilitate force concentration at specific sites within the lesion [3,5,75]. Several studies have demonstrated improved lesion preparation compared with conventional balloon angioplasty, particularly in moderately calcified lesions [3,5,75].

These devices may be particularly useful in lesions characterized by superficial calcium and limited calcium arc identified on OCT. However, their effectiveness may be reduced in the presence of extensive circumferential calcium or large calcified nodules [19,75].

#### 6.2.3. Ultra-High-Pressure Balloons

Ultra-high-pressure balloons represent another option for resistant calcified lesions. These devices can achieve inflation pressures exceeding 30 atmospheres and may produce calcium fractures that improve lesion compliance [69]. OCT studies have demonstrated that successful calcium fracture is strongly associated with improved stent expansion and procedural success [76].

Although effective in selected cases, ultra-high-pressure balloons should be used cautiously because excessive inflation pressures may increase the risk of vessel injury, perforation, or dissection [76].

### 6.3. Rotational Atherectomy

RA remains one of the most established techniques for the treatment of severely calcified coronary lesions [69,73,77]. The system utilizes a diamond-coated burr rotating at high speed to selectively ablate rigid calcific tissue while preserving more elastic vessel structures [69,77].

RA is particularly useful when severe calcification prevents device delivery or adequate balloon expansion. By reducing superficial calcium burden and modifying lesion compliance, RA facilitates subsequent balloon dilation and stent implantation [3,5,69,74,77]. The 2026 Japanese expert consensus update identifies RA as an established strategy for severely calcified lesions that remain uncrossable or undilatable despite balloon-based preparation and provides contemporary recommendations regarding technique, device selection, and complication management [69].

In lesions containing calcified nodules, RA may effectively debulk protruding calcium and improve luminal geometry before stenting [69,77,78]. Consequently, RA is often considered one of the most effective treatment options for protruding nodular calcium identified by IVUS or OCT [3,70,73]. This may be particularly advantageous in eruptive calcified nodules, where focal luminal protrusion represents a major obstacle to adequate stent expansion [30,31,49,78].

However, RA primarily modifies superficial calcium and may not adequately fracture deeper calcific plates [69,77]. Procedural complications such as slow-flow, burr entrapment, distal embolization, and coronary perforation remain important limitations [69,77].

### 6.4. Orbital Atherectomy

OA represents an alternative atherectomy-based strategy utilizing an eccentrically mounted diamond-coated crown. Unlike RA, which rotates concentrically, orbital atherectomy generates an orbital sanding motion that increases the area of calcium modification as rotational speed increases [70,78].

Potential advantages of OA include the treatment of larger vessels with a single crown size and modification of long or circumferentially calcified segments. However, the ECLIPSE randomized trial did not demonstrate that routine OA increased post-PCI minimum stent area or reduced target vessel failure at 1 year compared with a balloon-based preparation strategy in lesions considered suitable for both approaches [70]. These findings support a selective rather than routine role for OA in balloon-crossable calcified lesions.

The 2026 ECLIPSE OCT substudy provides additional mechanistic information on the effects of OA and balloon angioplasty on calcium modification [71]. Nevertheless, the specific role of OA in imaging-defined calcified nodules remains incompletely established, and available evidence is less robust than that supporting RA.

Despite promising results, additional comparative studies are required to determine the optimal role of OA in imaging-defined calcified nodules and to establish its position relative to RA and IVL [70,71,72,78].

### 6.5. Excimer Laser Coronary Atherectomy (ELCA)

ELCA is a specialized plaque-modification technique that utilizes pulses of ultraviolet light to disrupt atherosclerotic tissue through photochemical, photothermal, and photomechanical mechanisms [72,79,80]. Unlike RA and OA, which primarily target calcified tissue through mechanical ablation, ELCA acts by vaporizing intracellular water and disrupting plaque components at a microscopic level [72,79,80].

Historically, ELCA has been used in a variety of complex coronary lesions, including chronic total occlusions, in-stent restenosis, underexpanded stents, thrombotic lesions, and heavily calcified plaques resistant to conventional balloon angioplasty [79,80]. Its ability to modify both thrombotic and fibrocalcific components makes ELCA particularly attractive in selected lesions with mixed morphology.

In calcified lesions, ELCA alone generally produces less extensive calcium modification than RA, OA, or IVl. However, laser-induced microfractures and plaque disruption may facilitate subsequent balloon expansion and improve device deliverability [72,79,80,81]. Accordingly, ELCA is often used as an adjunctive rather than a standalone calcium-modification strategy.

The combination of ELCA with contrast injection (“laser contrast technique”) has been proposed as a strategy for resistant calcified lesions and severely underexpanded stents. The photomechanical effect generated by laser activation in the presence of contrast media may enhance plaque disruption and facilitate lesion expansion [81]. However, this approach requires considerable operator experience because of the increased risk of vessel injury and perforation.

Contemporary evidence includes the randomized ROLLER COASTR-EPIC22 comparison of RA, IVL, and ELCA, as well as real-world data from the ACCELERATE registry [72,79]. Nevertheless, evidence supporting the use of ELCA specifically in calcified nodules remains limited and is derived mainly from observational studies and case series. In contemporary practice, ELCA is therefore considered a specialized technique for selected lesions in which conventional calcium-modification strategies have failed or cannot be delivered [72,79,80,81]. Its use in calcified nodules should be individualized and guided by intravascular imaging findings.

### 6.6. Intravascular Lithotripsy (IVL)

IVL has rapidly emerged as one of the most important advances in the treatment of calcified coronary lesions. Adapted from extracorporeal lithotripsy used in urology, IVL delivers localized sonic pressure waves through a balloon-based catheter to selectively fracture vascular calcium while minimizing injury to surrounding soft tissues [26,27,82].

The mechanism of IVL differs fundamentally from that of atherectomy. Rather than removing calcium, IVL creates fractures within both superficial and deep calcific deposits, thereby increasing vessel compliance and facilitating subsequent stent expansion. This ability to modify deep calcium represents a major advantage over traditional atherectomy techniques, which primarily affect superficial calcium layers [26,27,60,82]. Consequently, IVL has become particularly attractive for lesions characterized by extensive circumferential calcium and deep calcific plates identified by IVUS or OCT.

OCT studies have provided important mechanistic insights into IVL-mediated calcium modification. Following IVL treatment, OCT frequently demonstrates multiple calcium fractures extending through both superficial and deep calcific plates. The presence, depth, and number of calcium fractures have been strongly associated with improved lesion compliance, greater stent expansion, and superior procedural outcomes [26,27,60,82]. These observations have established OCT as the preferred modality for evaluating the effectiveness of IVL-mediated calcium modification.

Several prospective studies, including the DISRUPT CAD program, have demonstrated high procedural success rates and favorable safety profiles for IVL in severely calcified coronary lesions [26,27]. Across these studies, IVL consistently improved stent expansion while maintaining low rates of perforation, slow-flow phenomena, and distal embolization. The ability of IVL to generate both superficial and deep calcium fractures distinguishes it from most other calcium-modification technologies and likely explains its favorable impact on stent expansion and procedural success [26,27]. As a result, IVL has increasingly been adopted as a first-line calcium-modification strategy for complex calcified lesions [3]. More recent evidence includes the randomized ROLLER COASTR-EPIC22 trial comparing IVL with RA and ELCA, as well as contemporary OCT-guided studies evaluating calcium fracture and procedural optimization [60,72].

The role of IVL in calcified nodules is particularly intriguing. Unlike RA, which debulks protruding calcium, IVL primarily acts by fracturing calcific structures within the vessel wall. Consequently, the effectiveness of IVL may vary according to calcified nodule morphology. Lesions characterized predominantly by deep sheet calcium with associated nodular protrusion may respond favorably to IVL because calcium fractures improve vessel compliance and facilitate stent expansion. In contrast, large eruptive calcified nodules with marked luminal protrusion may remain challenging because the protruding calcium mass itself is not removed by lithotripsy [26,30,31,78,82].

These observations have led to increasing interest in morphology-guided treatment selection. Current evidence suggests that protruding nodular calcium identified by OCT or IVUS may be more effectively treated with atherectomy-based strategies, whereas lesions dominated by deep circumferential calcium may be particularly suitable for IVL [3,26,31,78,82]. Accordingly, IVUS and OCT have become essential tools for selecting the most appropriate calcium-modification strategy.

Hybrid approaches combining atherectomy and IVL have also emerged as promising options for extremely complex calcified lesions. Hybrid RA–IVL (“RotaTripsy”) and OA–IVL (“OrbitalTripsy”) approaches integrate the debulking capabilities of atherectomy with the deep calcium-fracturing effects of IVL, thereby addressing both superficial and deep calcium components. Although current evidence remains limited, early observational studies suggest favorable procedural outcomes in lesions that would otherwise be difficult to treat using a single modality [72,83].

Importantly, IVL offers several practical advantages, including a relatively short learning curve, preservation of distal coronary flow during treatment, and a lower risk of distal embolization compared with atherectomy [3,26,27,82]. These characteristics have contributed to its rapid adoption in contemporary interventional practice and have positioned IVL as a central component of imaging-guided precision PCI for calcified coronary lesions. Furthermore, IVL may represent the most effective contemporary strategy for lesions characterized by deep circumferential calcium identified on OCT or IVUS, highlighting the transition from angiography-guided lesion preparation toward morphology-guided precision PCI based on intravascular imaging findings [3,26,31,78,82].

### 6.7. Stepwise IVUS/OCT-Guided Device Selection Based on Lesion Crossability and Calcium Morphology

The selection of an appropriate calcium-modification device should be based on a stepwise assessment of lesion crossability, balloon response, and calcium morphology rather than on angiographic severity alone. Table 2 summarizes an evidence-informed framework linking specific IVUS and OCT findings with the preferred initial strategy, alternative or rescue approaches, and the imaging endpoints required before stent implantation. This framework should be interpreted as a practical synthesis of the available evidence and expert consensus rather than as a prospectively validated treatment guideline [3,5,22,25,54].

The first decision point is whether the lesion can be crossed with an imaging catheter and an appropriately sized balloon. In balloon- or imaging-catheter-uncrossable lesions, or in lesions that remain undilatable despite high-pressure balloon inflation, an upfront facilitation or debulking strategy is generally required. RA is the preferred approach when severe superficial or nodular calcium prevents device passage or adequate balloon expansion. OA may be considered in selected anatomically suitable lesions, whereas ELCA may be useful in guidewire-crossable fibrocalcific lesions that remain uncrossable with a microcatheter or balloon, as well as in selected cases of resistant stent underexpansion [3,5,69,77,78,79,80,81].

When the lesion is balloon-crossable, IVUS or OCT should be used to determine calcium arc, thickness, length, depth, circumferential distribution, luminal protrusion, calcified nodule morphology, and the response to initial balloon dilatation. Lesions with limited superficial calcium, a relatively small calcium arc, and no marked nodular protrusion may initially be treated with a non-compliant, scoring, or cutting balloon. An ultra-high-pressure balloon or IVL may be considered when the initial balloon response remains inadequate [3,5,19,74,75,76].

Lesions dominated by thick, deep, or circumferential calcium are generally better suited to IVL, provided that the IVL balloon can be delivered across the lesion. IVL modifies both superficial and deep calcium by generating fractures that improve vessel compliance without removing calcium. When a coexisting superficial component prevents device passage, RA or OA may first be required to facilitate delivery of the IVL balloon [3,5,26,27,60,69,70,71,77,78,82].

Protruding nodular calcium and calcified nodules require separate consideration. Marked luminal protrusion, an irregular surface, and focal mechanical resistance generally favor RA because it can modify or debulk the superficial protruding component. OA may be considered as an alternative in selected lesions, although the evidence supporting its use specifically in calcified nodules remains less robust. When substantial nodular protrusion coexists with thick deep or circumferential calcium, a combined strategy using atherectomy followed by IVL may be required to address both the superficial and deep calcific components [30,31,49,69,70,71,77,78,82,83].

The presence of surface disruption or thrombus in an eruptive calcified nodule should also be considered during procedural planning. Significant thrombus burden requires individualized management, and atherectomy should not be selected solely on the basis of the diagnostic label of a calcified nodule. Instead, treatment should be determined by the dominant mechanical obstacle, lesion crossability, thrombus burden, vessel anatomy, and device deliverability [17,30,31,49,78].

In calcium-related stent underexpansion, IVL may be considered when the balloon can be delivered within the underexpanded segment and persistent deep or circumferential calcium is identified. ELCA, including ELCA followed by IVL, may be considered as a rescue strategy in experienced centres for selected resistant lesions. In such cases, intravascular imaging should confirm the mechanism of stent underexpansion and exclude alternative causes before additional calcium modification is undertaken [79,80,81,82].

After any calcium-modification step, IVUS or OCT should be repeated to assess calcium fracture, lumen gain, residual nodular protrusion, lesion compliance, and device deliverability. Stent implantation should proceed only after adequate lesion preparation has been demonstrated. If preparation remains insufficient, escalation to an alternative or hybrid strategy should be considered, followed by repeat intravascular imaging [22,25,54,60].

To translate these principles into practical procedural decision-making, Table 2 links lesion crossability and specific IVUS/OCT calcium characteristics with the preferred initial strategy, alternative or rescue treatment, and the required re-imaging endpoints.

### 6.8. Proposed Imaging-Guided Algorithm for Calcified Coronary Lesions and Calcified Nodules

**Figure 4:**
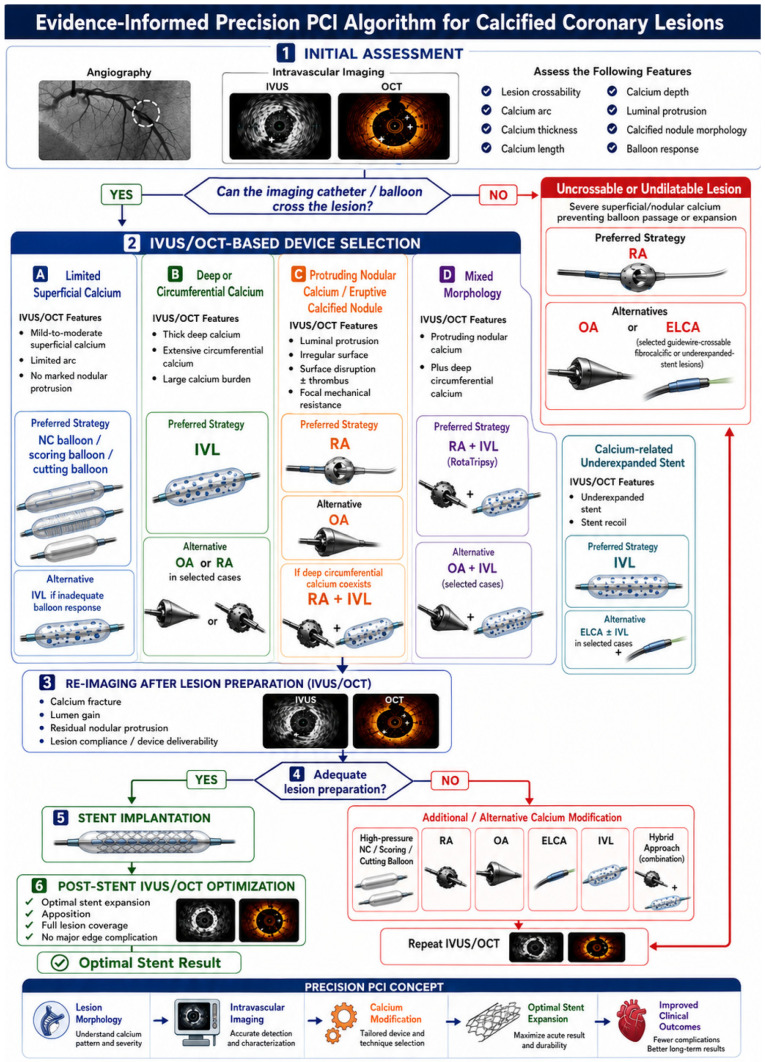
Evidence-Informed IVUS/OCT-Guided Precision PCI Algorithm for Calcified Coronary Lesions and Calcified Nodules. The proposed stepwise algorithm integrates lesion crossability, balloon response, calcium arc, thickness, length, depth, circumferential distribution, luminal protrusion, and calcified nodule morphology to guide device selection. Balloon- or imaging-catheter-uncrossable lesions generally require an upfront facilitation or debulking strategy using rotational atherectomy, orbital atherectomy, or, in selected lesions, excimer laser coronary atherectomy. Balloon-crossable lesions with limited superficial calcium may be treated with non-compliant, scoring, or cutting balloons, whereas deep or circumferential calcium generally favors intravascular lithotripsy. Markedly protruding or eruptive calcified nodules may favor rotational atherectomy, while mixed lesions combining nodular protrusion with deep circumferential calcium may require a hybrid atherectomy–lithotripsy strategy. Repeat IVUS or OCT after lesion preparation is recommended to assess calcium fracture, lumen gain, residual nodular protrusion, and lesion compliance before stent implantation. Post-stent imaging should confirm adequate stent expansion, apposition, lesion coverage, and the absence of major edge complications. The algorithm represents an evidence-informed synthesis and has not been prospectively validated as a formal treatment guideline; device selection should therefore be individualized according to lesion morphology, vessel anatomy, device deliverability, thrombus burden, operator experience, and local device availability [3,5,19,22,25,26,27,30,31,54,60,69,70,71,72,74,75,76,77,78,79,80,81,82,83]. Abbreviations: ELCA, excimer laser coronary atherectomy; IVL, intravascular lithotripsy; IVUS, intravascular ultrasound; NC, non-compliant balloon; OA, orbital atherectomy; OCT, optical coherence tomography; PCI, percutaneous coronary intervention; RA, rotational atherectomy.

Figure 4 translates the imaging–treatment relationships summarized in Table 2 into a stepwise procedural algorithm. The algorithm begins with angiographic assessment followed by IVUS or OCT whenever the lesion can be crossed with an imaging catheter. The initial evaluation should determine lesion crossability, calcium arc, thickness, length, depth, circumferential distribution, luminal protrusion, calcified nodule morphology, and the response to balloon dilatation [3,5,22,25,54].

If the lesion is uncrossable or remains undilatable, an upfront facilitation or debulking strategy should be selected, most commonly RA, with OA or ELCA reserved for selected anatomical and procedural settings. In balloon-crossable lesions, device selection is subsequently determined by the dominant imaging phenotype. Limited superficial calcium generally favors balloon-based modification; deep or circumferential calcium favors IVL; marked nodular protrusion or an eruptive calcified nodule may favor RA; and mixed lesions combining nodular protrusion with deep circumferential calcium may require a hybrid atherectomy–IVL approach [3,5,19,26,27,30,31,54,69,70,71,72,74,75,76,77,78,79,80,81,82,83].

A central component of the proposed algorithm is iterative intravascular imaging. Following the initial modification strategy, IVUS or OCT should be repeated to determine whether adequate calcium fracture, lumen gain, reduction in focal mechanical resistance, and improvement in lesion compliance have been achieved. If lesion preparation remains inadequate, an additional or alternative calcium-modification technique should be selected according to the residual imaging phenotype, followed by repeat imaging. This feedback loop is intended to avoid both premature stent implantation and unnecessary escalation of treatment [22,25,54,60].

Once satisfactory lesion preparation has been confirmed, stent implantation may proceed. Post-stent IVUS or OCT should then be used to assess minimal stent area, expansion, apposition, full lesion coverage, edge dissection, and residual protruding calcium. Additional post-dilatation or corrective treatment should be performed when predefined optimization targets have not been achieved [22,25,55,73].

The proposed algorithm is evidence-informed but has not been prospectively validated as a formal treatment guideline. Several of its branches—particularly those involving OA in calcified nodules, ELCA, and combined atherectomy–IVL strategies—remain supported predominantly by expert consensus, observational studies, or limited comparative evidence. Device selection should therefore be individualized according to lesion morphology, crossability, vessel dimensions, thrombus burden, side-branch anatomy, device availability, and operator experience [3,5,54,78,79,80,81,82,83].

## 7. Future Directions and Emerging Technologies

Despite substantial advances in intravascular imaging and calcium-modification devices, important uncertainties remain in the diagnosis and treatment of calcified coronary lesions and calcified nodules. Current practice still relies heavily on morphological assessment and operator experience, whereas lesion biology, natural history, and optimal treatment selection for specific calcific phenotypes remain incompletely defined [3,73].

Building on the diagnostic limitations discussed in Section 5.6, future clinical studies should prioritize the development and prospective validation of harmonized, modality-specific criteria for nodular calcium, non-eruptive calcified nodules, and eruptive calcified nodules [30,31,32,49,53]. Study protocols should explicitly report the imaging modality, diagnostic threshold, culprit or non-culprit lesion status, degree of luminal protrusion, surface morphology, presence of thrombus or surface disruption, and the underlying calcium architecture. Independent core-laboratory adjudication and, where feasible, validation against histopathological or multimodality imaging reference standards would improve reproducibility and enable more reliable comparisons of prevalence, prognosis, and treatment outcomes across studies.

In routine clinical practice, imaging-defined calcified nodules should not be treated as a uniform binary diagnosis. Until validated diagnostic criteria become available, operators should describe the individual morphological components of each lesion and base treatment decisions on calcium depth, arc, thickness, circumferential distribution, luminal protrusion, surface disruption, thrombus burden, vessel dimensions, and device deliverability rather than on the diagnostic label alone [3,5,30,31,49,54]. This morphology-based approach may reduce both overtreatment of relatively stable nodular calcium and insufficient lesion preparation in markedly protruding or eruptive calcified nodules.

Artificial intelligence (AI) and machine learning may support coronary calcium assessment by automating plaque segmentation, phenotyping, calcium quantification, and recognition of nodular protrusion on IVUS, OCT, and hybrid imaging datasets [22,84]. These tools could also support procedural planning by estimating the risk of stent underexpansion and identifying lesions likely to require advanced calcium modification [19,57,84]. However, their clinical value will depend on robust external validation, transparent model reporting, and successful integration into interventional workflows.

Multimodality platforms integrating IVUS, OCT, NIRS, angiography, and computational flow analysis may further improve the assessment of plaque structure, composition, biomechanics, and functional significance within a single procedural workflow [22,24,61,62,63,64,65,84]. By combining lesion morphology with plaque biology, these systems could support more precise risk stratification and individualized treatment selection before and during PCI [22,63,64,73].

Future imaging biomarkers should extend beyond calcium arc, thickness, and length to include calcium fracture patterns, microcalcification burden, biomechanical stress distribution, inflammatory activity, and interactions between calcific and lipid-rich plaque components [12,15,16,19,60,61,62,63,64,65,84]. These parameters may help distinguish relatively stable nodular calcium from lesions prone to thrombosis, rapid progression, or adverse procedural outcomes [17,30,31,32,49,51].

Device development is likely to focus on safer and more effective treatment of lesions combining protruding nodular calcium with extensive deep calcification [75,83]. Refinements in IVL, next-generation atherectomy systems, and morphology-guided hybrid strategies may expand therapeutic options. However, their optimal use should be defined by imaging-based lesion characteristics rather than by angiographic severity alone [3,71,75,77,82,83].

Several questions remain particularly important for calcified nodules, including their natural history, determinants of thrombotic transformation, mechanisms of calcium plate fracture, and factors driving progression from nodular calcium to eruptive calcified nodules [18,49,51]. It also remains unclear which imaging-defined nodules require aggressive calcium modification and which features identify lesions at the highest risk of stent underexpansion, restenosis, thrombosis, or target lesion failure [19,22,49,50].

Future trials should move beyond angiographic endpoints and evaluate morphology-guided treatment strategies. Recent randomized trials, including ECLIPSE and ROLLER COASTR-EPIC22, have strengthened the comparative evidence for calcium-modification strategies [70,72]. However, dedicated randomized trials enrolling patients with prospectively defined and imaging-confirmed calcified nodule phenotypes remain lacking. Future studies should therefore compare lesion-specific RA, OA, IVL, ELCA, and hybrid strategies in patients stratified according to calcified nodule morphology. Long-term studies incorporating detailed intravascular imaging will be essential to determine whether lesion-specific PCI improves clinical outcomes [3,22,54].

Ultimately, the integration of intravascular imaging, AI-assisted analysis, and morphology-guided calcium modification may shift treatment from generalized calcium management toward lesion-specific PCI for calcified nodules. This approach could improve procedural success, optimize stent expansion, reduce adverse cardiovascular events, and provide a more precise framework for the management of one of the most complex lesion subsets in contemporary coronary intervention [3,22,54,75,83].

## 8. Conclusions

Coronary calcification is an active biological and biomechanical process in which early inflammatory and osteogenic mineralization may progress to mature sheet calcium and, in selected lesions, to nodular or eruptive calcified phenotypes. Calcified nodules are heterogeneous lesions whose clinical relevance depends on luminal protrusion, surface disruption, thrombus formation, and their contribution to stent underexpansion and target lesion failure [8,10,12,17,18,20,30,31,32,49,50].

Contemporary intravascular imaging has become central to the assessment and treatment of calcified coronary lesions. OCT and IVUS provide complementary information on calcium morphology, vessel architecture, and procedural risk [22,25,54]. NIRS and hybrid imaging modalities add information on lipid-rich plaque composition and vulnerability [61,62,63,64,65]. Their integration enables morphology-guided calcium modification and supports a transition from angiography-guided PCI toward precision PCI [3,21,22,55,56,73].

Future research should harmonize pathological and imaging-based definitions of calcified nodules, clarify their natural history, and validate morphology-guided treatment algorithms in comparative clinical studies [30,31,32,53]. A deeper understanding of calcified nodule biology, combined with multimodality imaging, artificial intelligence-assisted plaque characterization, and contemporary calcium-modification technologies, may improve procedural success and long-term outcomes in patients with complex calcified coronary artery disease [54,73,78,82,83,84].

## Figures and Tables

**Table 1:** Advanced Coronary Calcific Phenotypes: Pathological Features, Imaging Characteristics, Clinical Significance, and Procedural Implications.

Feature	Sheet Calcium	Nodular Calcium	Calcified Nodule
Histopathological definition	Large confluent calcific plate embedded within a fibrocalcific plaque [18,20]	Protruding calcific mass without overt luminal surface disruption [48,49]	Fractured calcific plate with eruptive calcium, luminal protrusion, and frequently associated thrombus [13,17]
Dominant mechanism	Progressive mineral accumulation and plaque maturation [18,20,34,35]	Calcium plate fracture driven by biomechanical stress and repetitive vessel motion [17,18,30]	Advanced calcification with surface disruption, calcium eruption, thrombosis, and inflammatory activation [17]
Role of biomechanical stress	Limited contribution [18,20]	Important contributor to calcium fragmentation [17,18,30]	Central mechanism driving calcium plate disruption and luminal protrusion [17,18,30]
Luminal morphology	Flat or minimally protruding calcific surface [18,20]	Smooth protruding calcium extending into the lumen [48,49]	Irregular protruding calcium frequently associated with surface disruption and/or thrombus [13,17,48]
OCT characteristics	Homogeneous signal-poor region with sharply delineated borders [24,25]	Smooth protruding calcium with preserved luminal contour [48,51]	Irregular protruding calcium with disrupted surface and possible thrombus visualization [48,49,51]
IVUS characteristics	Extensive arc of calcium with acoustic shadowing [22,23,25]	Convex calcific protrusion with acoustic shadowing [22,23,25]	Convex protruding calcium; distinction from nodular calcium may be challenging [22,23,25]
Typical anatomical distribution	Diffuse fibrocalcific segments throughout the coronary tree [18,20]	Segments exposed to increased mechanical stress and vessel motion [18,28,30,32,49]	Frequently observed in the RCA, hinge-motion segments, and tortuous vessels [17,18,30,32,49]
Association with thrombosis	Rare [18,20]	Uncertain [51]	Frequently observed in pathological studies and culprit ACS lesions [13,17,48]
Association with ACS	Low [18,20]	Variable across studies [30,31,32,49,51]	Established but heterogeneous across pathology and imaging studies [17,34,48,51]
Relationship to plaque instability	Generally associated with plaque stabilization [18,20]	Intermediate and incompletely defined [51]	Frequently associated with plaque disruption and thrombogenic exposure [13,17]
Clinical significance	Vessel stiffening, impaired compliance, and altered coronary biomechanics [18,34,35]	Increased lesion complexity and procedural difficulty [49,51]	High-risk calcific phenotype associated with thrombosis, lesion instability, and adverse PCI outcomes [19,22,49,51]
Procedural implications	Balloon resistance and risk of stent underexpansion [3,19,22,25]	Device delivery difficulties and incomplete lesion preparation [19,22,49,50]	Severe lesion preparation challenges, stent underexpansion, restenosis, and target lesion failure [19,22,49,51]

Abbreviations: ACS, acute coronary syndrome; IVUS, intravascular ultrasound; OCT, optical coherence tomography; PCI, percutaneous coronary intervention; RCA, right coronary artery. Evidence basis: The pathological definitions and mechanistic comparisons were synthesized from histopathological and mechanistic studies [13,17,18,20], whereas imaging characteristics and clinical or procedural implications were based on intravascular imaging studies, consensus definitions, and PCI outcome studies [3,19,21,22,23,24,25,30,31,32,49,50,51,52,53]. Because pathological and imaging-based definitions are not fully concordant, the presented categories should be interpreted as related but not completely interchangeable calcific phenotypes.

**Table 2:** Evidence-Informed IVUS/OCT-Guided Device Selection for Calcified Coronary Lesions and Calcified Nodules.

Lesion Phenotype or Decision Point	Key IVUS/OCT Findings	Crossability and Balloon Response	Preferred Initial Strategy	Alternative or Escalation Strategy	Re-Imaging Endpoint or Procedural Caveat
**Un crossable or undilatable calcified lesion**	Severe superficial or nodular calcium; marked luminal narrowing; imaging catheter or balloon cannot cross; persistent balloon waist	Balloon or imaging-catheter uncrossable, or inadequate expansion despite high-pressure dilation	RA	OA in selected lesions; ELCA for selected guidewire-crossable but microcatheter- or balloon-uncrossable fibrocalcific lesions	Confirm device passage, lumen gain, and adequate balloon expansion before proceeding; repeat IVUS/OCT when technically feasible [3,5,69,77,78,79,80,81]
**Limited superficial calcium**	Mild-to-moderate superficial calcium; limited calcium arc; thin or short calcium; no marked nodular protrusion	Balloon-crossable with preserved or mildly impaired balloon expansion	NC balloon, scoring balloon, or cutting balloon	Ultra-high-pressure balloon or IVL if the initial balloon response is inadequate	Confirm improved lesion compliance and complete balloon expansion [3,5,19,74,75,76]
**High-burden superficial or circumferential calcium**	OCT: calcium arc >180°, thickness >0.5 mm, and/or length >5 mm; IVUS: calcium arc >270°, 360° superficial calcium, or a long calcified segment	Balloon-crossable but resistant to conventional dilation	IVL	RA or OA when a dominant superficial component or device-delivery limitation is present; hybrid treatment if modification remains inadequate	Confirm calcium fracture, lumen gain, and improved compliance before stenting [3,5,19,26,27,60,82]
**Deep sheet calcium**	Extensive deep calcium; deep circumferential calcific plates; large overall calcium burden; no dominant protruding mass	Balloon-crossable, with limited vessel compliance	IVL	Atherectomy may be used to facilitate IVL-balloon delivery when a coexisting superficial component prevents passage	OCT should demonstrate calcium fracture when visible; IVUS should confirm improved lumen geometry and balloon expansion [3,5,26,27,60,82]
**Protruding nodular calcium or non-eruptive calcified nodule**	Convex luminal protrusion; irregular or relatively preserved surface; focal mechanical resistance; no substantial thrombus	Balloon-crossable or focally undilatable	RA	OA in selected lesions; IVL when a deep circumferential component predominates and the balloon is deliverable	Assess residual protrusion, lumen gain, and lesion compliance after modification [30,31,49,69,77,78]
**Markedly protruding or eruptive calcified nodule**	Irregular luminal protrusion; disrupted surface; fractured underlying calcium; possible thrombus; severe focal resistance	Frequently undilatable; device passage may be impaired	RA when protruding superficial calcium is the dominant mechanical obstacle	RA + IVL when deep circumferential calcium coexists; OA may be considered in selected cases	Substantial thrombus burden requires individualized management; routine atherectomy should not be performed solely on the basis of the diagnostic label [17,30,31,49,78,82]
**Mixed morphology**	Protruding nodular calcium combined with thick deep or circumferential calcium	Incomplete response expected with a single modification modality	RA + IVL (RotaTripsy)	OA + IVL in selected cases	Repeat IVUS/OCT between treatment steps to determine whether further superficial debulking or deep calcium fracture is required [78,83]
**Calcium-related underexpanded stent**	Persistent stent underexpansion or recoil despite high-pressure balloon dilation; underlying deep or circumferential calcium	Stent lumen is balloon-crossable	IVL	ELCA ± IVL in experienced centres and selected resistant cases; other bailout techniques should be individualized	Confirm improvement in minimal stent area, apposition, and absence of major vessel injury [79,80,81,82]

Abbreviations: ELCA, excimer laser coronary atherectomy; IVL, intravascular lithotripsy; IVUS, intravascular ultrasound; NC, non-compliant balloon; OA, orbital atherectomy; OCT, optical coherence tomography; RA, rotational atherectomy. Evidence basis: The proposed device-selection framework was synthesized from contemporary expert consensus documents [3,5,53,69], randomized trials of intravascular imaging guidance and calcium modification [55,56,70,72], the ECLIPSE OCT substudy [71], OCT- and IVUS-based calcium assessment studies [19,23,52,59,60], and additional device-specific clinical evidence [26,27,69,70,71,72,74,75,76,77,78,79,80,81,82,83]. The framework is evidence-informed but has not been prospectively validated as a formal treatment guideline. Device selection should be individualized according to lesion crossability, calcium morphology, vessel anatomy, thrombus burden, device availability, and operator experience.

## Data Availability

No new data were created or analyzed in this study. Data sharing is not applicable to this article.

## Abbreviations

ACS	Acute Coronary Syndrome
AI	Artificial Intelligence
ALP	Alkaline Phosphatase
BMP2	Bone Morphogenetic Protein 2
CAC	Coronary Artery Calcification
CAD	Coronary Artery Disease
CKD	Chronic Kidney Disease
ELCA	Excimer Laser Coronary Atherectomy
EV	Extracellular Vesicle
FGF-23	Fibroblast Growth Factor 23
IL	Interleukin
IVL	Intravascular Lithotripsy
IVUS	Intravascular Ultrasound
LCBI	Lipid Core Burden Index
NC	Non-compliant Balloon
NIRS	Near-infrared Spectroscopy
OA	Orbital Atherectomy
OCT	Optical Coherence Tomography
PCI	Percutaneous Coronary Intervention
RA	Rotational Atherectomy
RCA	Right Coronary Artery;
ROS	Reactive Oxygen Species
RUNX2	Runt-related Transcription factor 2
TNF-α	Tumor Necrosis factor-alpha
VSMC	Vascular Smooth Muscle Cell
